# Mild COVID-19 induces early, quantifiable, persistent troponin I elevations in elder men

**DOI:** 10.3389/fcvm.2022.1053790

**Published:** 2022-12-01

**Authors:** Justus J. Bürgi, Matthias Rösslein, Oliver Nolte, Peter Wick, Regine Garcia Boy, Siegfried Stranders, Günter Dollenmaier, Karen Peier, Brigitte Nohynek, Aldo Fischer, Raphael Stolz, Michele Cettuzzi, Lukas Graf, Wolfgang Korte

**Affiliations:** ^1^Center for Laboratory Medicine, St. Gallen, Switzerland; ^2^Swiss Federal Laboratories for Materials Science and Technology, St. Gallen, Switzerland; ^3^Cantonal Corona Study Group, St. Gallen, Switzerland; ^4^Public Health Department, St. Gallen, Switzerland

**Keywords:** mild COVID-19, troponin, gender-dependent, age-dependent, longitudinal, cohort study

## Abstract

**Importance:**

Elderly patients, especially men, are at risk of increased morbidity from coronavirus disease 2019 (COVID-19). Long-term data on troponin I levels in longitudinal observational studies of outpatients with mild to moderate COVID-19 are scarce.

**Objective:**

This controlled cohort study aimed to evaluate the course of troponin I concentrations over a long period in convalescent COVID-19 outpatients with mild to moderate symptoms.

**Setting and participants:**

In this cohort study, individuals with PCR-confirmed, mild to moderate SARS-CoV-2 infection as well as control individuals with confirmed negative PCR and negative SARS-CoV-2 serology were included. Study visits were performed from April 2020 through July 2021 (initialized during the first wave of the corona pandemic in Switzerland). A study visit in patients comprised blood draws every week in the first month and additionally after 8 weeks. This course was repeated in patients observed long-term.

**Results:**

This study enrolled 278 individuals from the Canton of St. Gallen, Switzerland, aged 12–92 years (59.5% women), who had mild to moderate COVID-19 symptoms (outpatients only) and a diagnosis confirmed by positive RT-PCR. Fifty-four of the participants with confirmed SARS-CoV-2 infection were followed for 14 months with repeat cycles of the testing protocol. In addition, 115 symptomatic patients that were PCR and serology negative were enrolled in the same time period as a control group. In COVID-19 patients, low-level troponin I concentrations (cTnI) were significantly increased from baseline until week 9 after positive RT-PCR diagnosis in men older than 54 years [ΔcTnI = 5.0 ng/L (median); 95% CI 4.1–6.0; *p* = 0.02]. The troponin I concentration remained elevated throughout 14 months in men older than 54 years within the cohort with a prolonged observation period. This statistically significant change in troponin I concentration was not dependent on co-morbidities in this group. ALT, Creatinine, BNP, and D-Dimer values after convalescence did not differ in comparison to the control cohort.

**Conclusion:**

In this analysis of individuals with confirmed SARS-CoV-2 infection, hs troponin I levels of men aged 54 or older significantly increased after infection. They remained elevated for at least 14 months after diagnosis. This suggests the possibility of an ongoing, long-term, low-grade myocardial injury. Further studies with focus on elderly patients and a prolonged observational period are necessary to elucidate whether the phenomenon observed is associated with detectable structural changes to the heart muscle or is without further clinical consequences.

## Introduction

Cardiac involvement in severe COVID-19, the disease caused by severe acute respiratory syndrome coronavirus 2 (SARS-CoV-2), is common. Recent evidence suggests that increased troponin levels in patients with severe (1) COVID-19 are associated with an increased likelihood of cardiovascular disease, critical illness (2, 3), and death (4, 5). Patients with COVID-19 not only show troponin levels above the 99th percentile more frequently, but also show measurably increased levels of troponins (below the 99th percentile but above the limit of detection) more frequently (6–9).

Research has shown that evaluating troponin levels over time with the detection of progressive changes more reliably identifies myocardial damage as compared to measuring at a single time point using defined cut-off levels (10, 11). Moreover, changes in low range troponin concentrations were found to be associated with future heart failure (12) and coronary artery disease (CAD) (13), despite being measured below the 99th percentile of a normal cohort. These findings have recently been confirmed in a very large metaanalysis (14). Specifically, any increases in very low troponin I levels (i.e., <6 ng/L) over time are associated with an increased risk for cardiovascular events (15). In a pilot study, the highest efficacy to predict CAD was seen in patients with a troponin increase of 20% or more over time (16). These findings not only are relevant for cohorts of patients; but have been shown to be also applicable to singular cases (17). Thus, these observations provide evidence that even small increases in low (i.e., below the 99th percentile) troponin I levels over time are associated with the risk for an adverse clinical outcome. To the best of our knowledge, no prolonged longitudinal observations on troponin levels in mild COVID-19 cases over time have been published so far.

We here report our long-term results in patients with PCR confirmed SARS-CoV-2 infection and non-severe COVID-19.

## Materials and methods

### Study population

The multi-center cohort study (K2-study of the Canton of St. Gallen, Switzerland) as well as the control cohort (G2-study of the Canton of St. Gallen, Switzerland) were registered in the Swiss COVID-19 database^1^ and approved by the regional ethics committee (ID: 2020-00941 and ID: 2020-01063). We reached out to potential participants through the distribution of flyers to all general practitioners in the Canton of St. Gallen, through a call for participation in social media and through the identification through the public health department database. The criteria for participation in the study were a positive SARS-CoV-2 PCR result (or, for the control group, a negative PCR result in addition to a negative SARS-CoV-2 serology), informed consent and the ability to contact and visit outpatient test centers on their own. Upon inclusion into the study, a questionnaire was to be completed. The control cohort (*n* = 115) was composed of participants with COVID-19 like symptoms but with a negative PCR result from a nasopharyngeal swab and with negative SARS-CoV-2 serology [anti-SARS-CoV-2 (spike) IgA/IgG]. PCR positive participants than had multiple study visits with blood draws. The first cycle of serial blood draws (blood draws every week in the first month and then one blood draw after another 4 weeks in the second month, a total of 5 visits per cycle) in the PCR-positive cohort (*n* = 278, entire cohort) took place between April 2020 and July 2020. Participants were offered to repeat cycles of repeated blood draws later on. Fifty-four of the individuals repeatedly participated in up to three study visit cycles of blood draws at various time points (long-term sub cohort), effectively providing detailed longitudinal data over a duration of more than 1 year. Detailed cohort characteristics as well as co-morbidities are described in Table 1.

**TABLE 1:** Cohort characteristics.

Cohort characteristics	Entire cohort	Long-term sub cohort	Control cohort
Number of individuals	278	54	115
Age (years)	12.0–91.2 (Median = 51.2, IQR = 25.8)	24.8–91.2 (Median = 55.8, IQR = 13.9)	18–81 (Median = 51, IQR = 23.5)
Female	59.5% (166/278)	40.7% (22/54)	71.3% (82/115)
Sample collection dates	April 2020–July 2020	April 2020–June 2021	April 2020– February 2021
Weeks after PCR until first visit	1–40 (Median = 6, IQR = 4)	1–9 (Median 6, IQR = 3, *n* = 54)	0–48 (Median 9, IQR = 6, *n* = 115)
Number of study visits*			
Total sera count	2016	741	115
1–5 visits	68.4% (1378/2016)	36.2% (268/741)	100% (115/115)
6–10 visits	21.5% (434/2016)	35.8% (265/741)	N/A
11–15 visits	10.1% (204/2016)	28.0% (208/741)	N/A
**Co-morbidities**			
Hypertension	18.8% (52/277)	18.9% (10/53)	7.3% (8/110)
Cancer	3.6% (10/276)	3.8% (2/53)	4.5% (5/110)
Diabetes	4.0% (11/277)	5.7% (3/53)	2.7% (3/110)
Pulmonary diseases	9.7% (27/277)	15.1% (8/53)	3.6% (4/110)
Immune system disorders	3.2% (9/277)	0% (0/53)	7.3% (8/110)
Coronary-/artery diseases	8.0% (22/276)	9.6% (5/52)	4.5% (5/110)
Smokers	56.1% (156/278)	5.7% (3/53)	15.4% (17/110)
Former smokers	31.4% (87/277)	32.1% (17/53)	35.4% (39/110)
Non-smokers	10.8% (30/277)	62.3% (33/53)	47.2% (52/110)

Summary of demographic characteristics, number of study visits, and co-morbidities are described. Note that the control cohort also presented COVID-19 like symptoms, but with negative PCR diagnosis and SARS-CoV-2 serology. IQR, interquartile range.

*The control cohort had only one study visit.

### Measurements

At ZLM, swabs for PCR (Copan ESwap^®^) were collected from the nasopharynx. Samples were extracted using Molgen PurePrep Extraction Kit and the IDEAL96 extraction robot, followed by RT-PCR targeting sequences of the SARS-CoV-2 E-gene, based on published protocols (18, 19). Blood draws were obtained from an antecubital vein into vacuum tubes (BD 367955). Serum was immediately used or stored at −80°C until further use. High sensitive troponin I was measured with the (Beckman Coulter Switzerland, Nyon, Switzerland) Access high sensitive troponin I assay (limit of detection and limit of quantification both at 2.3 ng/L) on a UniCel DxI 800 (Beckman Coulter Switzerland, Nyon, Switzerland). All determinations were performed with the same reagent batch. Creatinine and alanine aminotransferase (ALT) levels were measured on an AU 5800 (Beckman Coulter Switzerland, Nyon, Switzerland). D-Dimer (D-Dimer HS 500, IL) was measured on an ACL TOP 750 (II, AxonLab, Baden, Switzerland) and brain natriuretic peptide (Quidel Ireland Ltd., Galway Ireland) was measured on a UniCel DxI 800 (Beckman Coulter Switzerland, Nyon, Switzerland). All assays were performed according to the recommendations of the manufacturer in an accredited laboratory (ZLM St. Gallen, ISO 17025). Quality control was performed following the manufacturer’s instructions and on each day of testing.

### Statistical analysis

*P*-values have been calculated using an unpaired, two-sided *t*-test with independent variances, or the Wilcoxon rank sum, respectively the Mann–Whitney test. *P*-values of <0.05 were considered statistically significant. Statistical definitions, analysis and visualization were based on or performed with software R, using the implemented statistical tests and the packages “tidyverse” and “ggplot2” (20). Data are presented as median with interquartile range (IQR).

## Results

### Enrollment

Two hundred and seventy-eight participants (entire cohort), aged 12.0–91.2 years (Median = 51.2, IQR = 25.8) who had a positive SARS-CoV-2 RT-PCR diagnosis were enrolled (Table 1); 26 individuals that had COVID-19 and a need for hospitalization were excluded. The entire cohort (*n* = 278) contained 166 (59.5%) women and 112 (40.5%) men. The first visit was conducted at a median of 6 weeks (range 1–40, IQR = 4) after positive PCR diagnosis. In total, 2016 sera were collected for further analysis. Among the 278 participants, 54 participants aged 24.8–91.2 (Median = 55.8, IQR = 13.9) repeated the study course three times. This long-term sub cohort (*n* = 54) consisted of 22 (40.7%) women and 32 (59.3%) men, whereof 20 (37%) men were older than 54 years. If individuals were vaccinated during the study course, only data from visits before the first vaccination took place were included (66 sera of 24 participants were excluded).

A control cohort of 115 participants (control cohort) aged 18.0–81.0 (Median = 51.0, IQR = 23.5) was enrolled in parallel (Table 1); 11 participants were excluded (6 because of missing SARS-CoV-2 PCR data and 5 because of missing troponin values). The control cohort (*n* = 115) consisted of 82 (71.3%) women and 33 (28.7%) men. Due to the recruitment design, all control individuals were also symptomatic. First visit for blood draws was conducted at a median of 9 weeks (range 0–41, IQR = 6) after negative PCR diagnosis.

### Troponin concentrations

**FIGURE 1:**
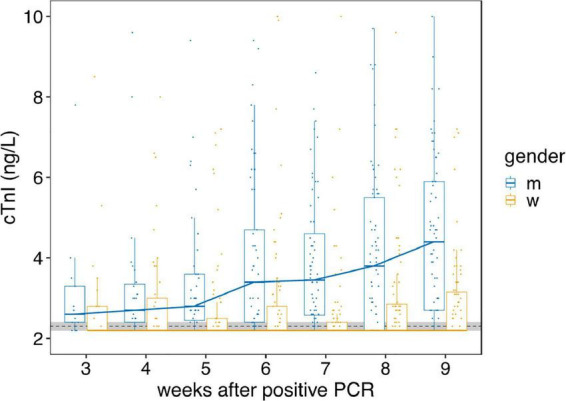
Gender-dependent course of hs troponin I concentrations from week 3 to 9. The course of hs troponin I concentrations (cTnI) from week 3 to 9 [high sensitive (Beckman Coulter Switzerland, Nyon, Switzerland) assay] after mild to moderate SARS-CoV-2 infections in women (w, yellow) and men (m, blue) are displayed. The overall change of cTnI over 9 weeks is highly significant in men (*p* < 0.0001), but not in women. Black dotted line indicate the limit of detection and limit of quantification, both at 2.3 ng/L. Horizontal bold lines indicate median values; boxes indicate quartiles 1 and 3; whiskers indicate 1.5 × IQR confidence intervals (CI). Gray shaded region indicate the 95% CI of the cTnI of the control cohort (*n* = 115). Each point represents a single measurement.

Median high sensitive troponin I concentration (cTnI) in the entire cohort (*n* = 278) over the whole study duration of 14 months was 2.7 ng/L (95% CI 2.0–3.4 ng/L). Longitudinal gender-specific analysis of the cTnI results showed a significant increase from week 3 (2.65 ng/L, 95% CI 1.3–4.7 ng/L) to week 9 (4.65 ng/L, 95% CI 4.0–5.4 ng/L) after infection in men (Figure 1, *p* < 0.0001). The cTnI level of men at week 9 is significantly higher compared to men with a negative SARS-CoV-2 PCR and serology (median cTnI 2.8 ng/L, 95% CI 2.47–3.17 ng/l, *p* < 0.0001) and as well-compared to the baseline value of the entire positive cohort (2.7 ng/L, 95% CI 2.0–3.4 ng/L). In contrast, no significant increase of cTnI was detected in females during the first 9 weeks after SARS-CoV-2 infection (Figure 1).

### Troponin levels up to 57 weeks post positive PCR

**FIGURE 2:**
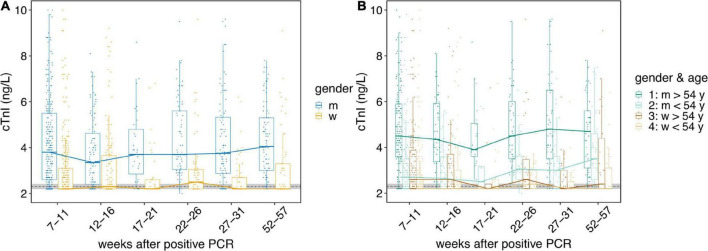
The course of hs troponin I concentrations over a period of more than 1 year. The course of hs troponin I concentrations (cTnI) over a period of more than 1 year [high sensitive (Beckman Coulter Switzerland, Nyon, Switzerland) assay] after mild to moderate SARS-CoV-2 infections are displayed. Black dotted line indicate the limit of detection and limit of quantification, both at 2.3 ng/L. Horizontal bold lines indicate median values; boxes indicate quartiles 1 and 3; whiskers indicate 1.5 × IQR confidence intervals (CI). Gray shaded region indicate the 95% CI of the cTnI of the control cohort (*n* = 115). Each point represents a single measurement. **(A)** Gender-dependent cTnI. The course of cTnI from week 7 to 57 in women (w, yellow) and men (m, blue) are displayed. **(B)** Course of cTnI over time split into four categories: 1: men older than 54 years (dark green); 2: men younger than 54 years (light green); 3: women older than 54 years (brown); 4: women younger than 54 years (beige). Difference in cTnI from older (>54 y, *n* = 20) men to the control cohort is highly significant (*p* > 1.6 10^–8^).

Extending the observation period to 57 weeks (long-term sub cohort, *n* = 54) revealed also no significant change in cTnI in women. However, median cTnI in men remained persistently increased (after the initial significant cTnI increase) over the entire observation period (Figure 2A). Further analysis disclosed a gender and age dependency of the changes observed (Figure 2B). Only men older than 54 years showed significant increased cTnI values over the entire period of more than 1 year. Men younger than 54 years did not present elevated cTnI values (Figure 2B).

### Evaluation of potential confounders of troponin I concentration results

As recently shown in an animal model (21), cardiac troponins are renally and hepatically cleared. We thus evaluated markers of kidney and liver function in the cohort study. Although alanine aminotransferase (ALT) levels showed the expected gender difference (22) in men (median 26.4 U/l) and women (median 17.6 U/l), ALT values did not change over time in neither gender (p for difference > 0.12 in men and *p* > 0.33 in women). Also, ALT values in neither women (*p* > 0.23) nor men (*p* > 0.12) were different from those observed in the control cohort. The same circumstances were true for creatinine: values were expectedly (23) higher in men (median 82.9 μmol/l) than women (median 65.6 μmol/l); but they were again unchanged over time in both genders (men *p* > 0.37, women *p* > 0.24). Again, no significant differences to the control cohort were seen (men *p* > 0.19, women *p* > 0.15). The same was true for D-Dimer and brain natriuretic peptide (BNP): D-Dimer values (median in men 0.456 mg/l; median in women 0.466 mg/l) were unchanged over time in comparison to the control cohort (men *p* > 0.65, women *p* > 0.72); BNP values (median in men 25.4 ng/l; median in women 33.4 ng/l) showed a similar behavior and were not significantly altered in comparison to the control cohort (men *p* > 0.83, women *p* > 0.98).

### Co-morbidities and troponin I concentration

**FIGURE 3:**
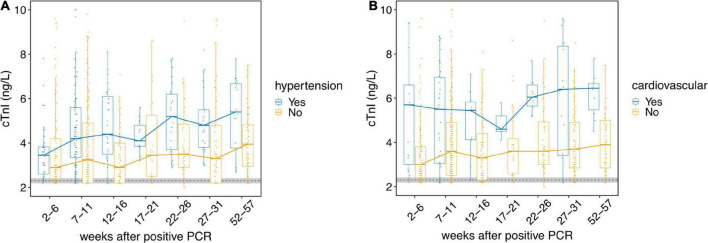
The course of hs troponin I concentrations according to different co-morbidities. The course of hs troponin I concentrations (cTnI) over a period of more than 1 year [high sensitive (Beckman Coulter Switzerland, Nyon, Switzerland] assay) after mild to moderate SARS-CoV-2 infections depending on different co-morbidities is presented. Black dotted line indicate the limit of detection and limit of quantification, both at 2.3 ng/L. Horizontal bold lines indicate median values; boxes indicate quartiles 1 and 3; whiskers indicate 1.5 × IQR confidence intervals (CI). Gray shaded region indicate the 95% CI of the cTnI of the control cohort (*n* = 115). Each point represents a single measurement. **(A)** The cohort is split by data from the survey indicating hypertension (Yes, blue) or no hypertension (No, yellow). The course of cTnI over time is presented. **(B)** The cohort is split by data from the survey indicating an occurrence of a known cardiovascular disease (Yes, blue) or no cardiovascular disease (No, yellow). The course of cTnI over time is presented.

Potential associations between various significant conditions of the patients (cancer, diabetes, pulmonary disease, cardiovascular disease, hypertension, immune suppression, smoking, and the intake of medications) and cTnI levels were evaluated. Only cardiovascular disease and hypertension were associated with cTnI (Figure 3), all other conditions did not associate with cTnI.

## Discussion

To the best our knowledge, so far neither longitudinal data on troponin changes for (out)patients with non-severe COVID-19 have been reported; nor have reports included longitudinal observations for more than 1 year in comparison to a control cohort (6, 24).

The main results from our study show for the first time that mild to moderate courses of COVID-19 can be associated with an early increase in cardiac troponin levels, indicating myocardial damage. Moreover, in men with mild to moderate COVID-19 and which are more than 54 years old, cTnI levels remained elevated during an observation period of up to 14 months.

There is a known, stable gender-difference (25) in cTnI; however, this can not explain the significant increase observed during the first 9 weeks after SARS-CoV-2 infection in men older than 54 years. Also, renal and hepatic dysfunction (with potentially reduced troponin clearance) was ruled out. Thus, it seems that the significant increase in cTnI in the weeks after SARS-CoV-2 infection reflect mild, but ongoing myocardial damage secondary to the SARS-CoV-2 infection.

It has been shown that myocardial damage occurs in about 20% of patients with severe COVID-19 (26). In such circumstances, even mild increases of cTnI (well below the 99th percentile) are associated with a higher prevalence of cardiac and non-cardiac complications, and a higher mortality (8, 27). In 2,700 patients with severe COVID-19, signs of myocardial injury were common and usually associated with mild elevations in troponin concentration (28). It seems likely that this myocardial injury is the result of a combination of cytokine release, inflammation, microvascular damage, and a resulting supply/demand imbalance (28). With this understanding, it is conceivable that different immunological responses lead to different clinical susceptibilities of myocardial damage. And early data from our cohort have indeed revealed a gender difference in early immunological response after SARS-CoV-2 infection (29).

Subclinical left ventricular strain in about 30% of COVID-19 recovered individuals has been reported (30, 31). In a small study on outpatients in cardiologic care (32), cardiac involvement after outpatient recovery from COVID-19 was reported in 71% of the participants. Our study, however, describes a variety of otherwise unselected patients and seems to identify a risk group.

Sudden cardiac death after otherwise uneventful COVID-19 has been reported (33). This is in line with earlier immunological results from our cohort, potentially suggesting persistence of the virus (34). In fact, COVID-19 seems able to induce enduring myocardial damage (35) and post-mortem analyses have shown quantifiable and persistent SARS-CoV-2 RNA in multiple organs, including the heart (36). This might also explain cases of myocarditis in COVID-19 patients (4).

Given the current knowledge, it seems that also low-level troponin changes are associated with an increased risk for cardiovascular disease during the later course (37). Our study evidences that such changes can start early on after mild to moderate COVID-19; and can continue to be detectable for multiple months thereafter. It remains to be elucidated how recurring infections, that are meanwhile repeatedly seen, will influence the picture.

Our study has some potential limitations: this is a relatively small cohort study based on SARS-CoV-2 PCR positivity in outpatients with a history of mild COVID-19. However, a strict protocol was in place and patients have been sampled repeatedly in designated COVID test centers with specifically dedicated medical personnel, making it unlikely that the inclusion and sampling procedures were biased.

Also, no further cardiac studies (e.g., ECG, echocardiography) were performed, which, at the time, was impossible due to the pandemic situation with reduction of non-emergent outpatient services. Nonetheless, this approach allowed us to repeatedly collect reliable data during the first two pandemic waves including data from a control cohort, which is a novelty.

## Conclusion

In this controlled cohort study (with the control cohort observed during the same pandemic wave) in patients with SARS-CoV-2 infection and mild to moderate COVID-19, troponin I levels of men aged 54 or older are significantly increased early after infection and continue to be increased for as long as 14 months thereafter. Our results support the notion of an early and continuing cardiac involvement (in men older than 54 years) after mild to moderate COVID-19.

Further studies with focus on persons older than 54 years with an elongated observation period are necessary to elucidate whether the phenomenon observed might be associated with detectable long-term structural changes to the heart, is reversible or might remain without long term clinical consequences.

## Data availability statement

The raw data supporting the conclusions of this article will be made available by the authors, without undue reservation.

## Ethics statement

The studies involving human participants were reviewed and approved by the Ethikkommission Ostschweiz (EKOS). Written informed consent to participate in this study was provided by the participants’ legal guardian/next of kin.

## Author contributions

WK, MR, and JB: full access to all of the data in the study and took responsibility for the integrity of the data and the accuracy of the data analysis. ON, JB, and WK: concept and design. JB, MR, RG, SS, GD, BN, AF, RS, MC, PW, and WK: acquisition, analysis, and interpretation of data. MR, PW, and JB: statistical analysis. PW and WK: obtained funding. JB, ON, MR, RG, SS, KP, BN, AF, RS, MC, LG, and WK: administrative, technical, and material support. JB, PW, and WK: supervision. JB and WK: drafting of the manuscript. All authors contributed to the article and approved the submitted version.

## Footnotes

1
https://swissethics.ch/covid-19/approved-projects

## References

[1] Sandoval Y Januzzi JL Jr. Jaffe AS. Cardiac troponin for assessment of myocardial injury in COVID-19: JACC review topic of the week. *J Am Coll Cardiol.* (2020) 76:1244–58. 10.1016/j.jacc.2020.06.068 32652195 PMC7833921

[2] Petrilli CM Jones SA Yang J Rajagopalan H O’Donnell L Chernyak Y Factors associated with hospital admission and critical illness among 5279 people with coronavirus disease 2019 in New York City: prospective cohort study. *BMJ.* (2020) 369:m1966. 10.1136/bmj.m1966 32444366 PMC7243801

[3] Shah P Doshi R Chenna A Owens R Cobb A Ivey H Prognostic value of elevated cardiac troponin i in hospitalized covid-19 patients. *Am J Cardiol.* (2020) 135:150–3. 10.1016/j.amjcard.2020.08.041 32861733 PMC7452835

[4] Guo T Fan Y Chen M Wu X Zhang L He T Cardiovascular implications of fatal outcomes of patients with coronavirus disease 2019 (COVID-19). *JAMA Cardiol.* (2020) 5:811–8. 10.1001/jamacardio.2020.1017 32219356 PMC7101506

[5] Du RH Liang LR Yang CQ Wang W Cao TZ Li M Predictors of mortality for patients with COVID-19 pneumonia caused by SARS-CoV-2: a prospective cohort study. *Eur Respir J.* (2020) 55:2000524. 10.1183/13993003.00524-2020 32269088 PMC7144257

[6] Velavan TP Kuk S Linh LTK Lamsfus Calle C Lalremruata A Pallerla SR Longitudinal monitoring of laboratory markers characterizes hospitalized and ambulatory COVID-19 patients. *Sci Rep.* (2021) 11:14471. 10.1038/s41598-021-93950-x 34262116 PMC8280222

[7] Puntmann VO Carerj ML Wieters I Fahim M Arendt C Hoffmann J Outcomes of cardiovascular magnetic resonance imaging in patients recently recovered from coronavirus disease 2019 (COVID-19). *JAMA Cardiol.* (2020) 5:1265–73. 10.1001/jamacardio.2020.3557 32730619 PMC7385689

[8] Caro-Codón J Rey JR Buño A Iniesta AM Rosillo SO Castrejon-Castrejon S Characterization of myocardial injury in a cohort of patients with SARS-CoV-2 infection. *Med Clin (Engl Ed).* (2021) 157:274–80. 10.1016/j.medcli.2021.02.001 34568576 PMC8451250

[9] Mueller C Giannitsis E Jaffe AS Huber K Mair J Cullen L Cardiovascular biomarkers in patients with COVID-19. *Eur Heart J Acute Cardiovasc Care.* (2021) 10:310–9. 10.1093/ehjacc/zuab009 33655301 PMC7989520

[10] Ungerer JP Tate JR Pretorius CJ. Discordance with 3 cardiac troponin I and T Assays: implications for the 99th percentile cutoff. *Clin Chem.* (2016) 62:1106–14. 10.1373/clinchem.2016.255281 27335076

[11] Lippi G Lavie CJ Sanchis-Gomar F. Cardiac troponin I in patients with coronavirus disease 2019 (COVID-19): evidence from a meta-analysis. *Prog Cardiovasc Dis.* (2020) 63:390–1. 32169400 10.1016/j.pcad.2020.03.001 PMC7127395

[12] Stelzle D Shah ASV Anand A Strachan FE Chapman AR Denvir MA High-sensitivity cardiac troponin I and risk of heart failure in patients with suspected acute coronary syndrome: a cohort study. *Eur Heart J Qual Care Clin Outcomes.* (2018) 4:36–42. 29045610 10.1093/ehjqcco/qcx022 PMC5805120

[13] Olson F Engborg J Gronhoj MH Sand NP Lambrechtsen J Steffensen FH Association between high-sensitive troponin I and coronary artery calcification in a Danish general population. *Atherosclerosis.* (2016) 245:88–93. 10.1016/j.atherosclerosis.2015.12.017 26714045

[14] Willeit P Welsh P Evans JDW Tschiderer L Boachie C Jukema JW High-sensitivity cardiac troponin concentration and risk of first-ever cardiovascular outcomes in 154,052 participants. *J Am Coll Cardiol.* (2017) 70:558–68. 10.1016/j.jacc.2017.05.062 28750699 PMC5527070

[15] White HD Tonkin A Simes J Stewart R Mann K Thompson P Association of contemporary sensitive troponin I levels at baseline and change at 1 year with long-term coronary events following myocardial infarction or unstable angina: results from the LIPID study (long-term intervention with pravastatin in ischaemic disease). *J Am Coll Cardiol.* (2014) 63:345–54. 10.1016/j.jacc.2013.08.1643 24140630

[16] Fabregat-Andres O Valle-Munoz A Corbi-Pascual M Ferrando-Beltran M Lucas-Inarejos E Ridocci-Soriano F. Diagnostic implication of the percentage change in troponin I in normal range in patients with suspected unstable angina. *Rev Esp Cardiol (Engl Ed).* (2012) 65:674–6. 10.1016/j.recesp.2011.10.012 22277910

[17] Quiroz R Joseph L Sam F. Serial troponin-I measurement as a diagnostic and therapeutic tool in chronic myocarditis. *J Heart Lung Transplant.* (2010) 29:820–2. 10.1016/j.healun.2010.03.006 20417129 PMC2904438

[18] Corman VM Landt O Kaiser M Molenkamp R Meijer A Chu DK Detection of 2019 novel coronavirus (2019-nCoV) by real-time RT-PCR. *Eurosurveillance.* (2020) 25:2000045. 10.2807/1560-7917.ES.2020.25.3.2000045 31992387 PMC6988269

[19] Rüfenacht S Gantenbein P Boggian K Flury D Kern L Dollenmaier G Remdesivir in Coronavirus Disease 2019 patients treated with anti-CD20 monoclonal antibodies: a case series. *Infection.* (2022) 50:783–90. 10.1007/s15010-022-01821-y 35426564 PMC9010446

[20] Wickham H Averick M Bryan J Chang W McGowan LD François R Welcome to the tidyverse. *J Open Source Softw.* (2019) 4:1686.

[21] Muslimovic A Friden V Tenstad O Starnberg K Nystrom S Wesen E The Liver and Kidneys mediate clearance of cardiac troponin in the rat. *Sci Rep.* (2020) 10:6791. 10.1038/s41598-020-63744-8 32322013 PMC7176693

[22] Trambas C Pickering JW Than M Bain C Nie L Paul E Impact of high-sensitivity troponin I testing with sex-specific cutoffs on the diagnosis of acute myocardial infarction. *Clin Chem.* (2016) 62:831–8. 10.1373/clinchem.2015.252569 27117468

[23] Culleton BF Larson MG Evans JC Wilson PWF Barrett BJ Parfrey PS Prevalence and correlates of elevated serum creatinine levels: the Framingham heart study. *Arch Intern Med.* (1999) 159:1785–90. 10.1001/archinte.159.15.1785 10448783

[24] Petersen EL Goßling A Adam G Aepfelbacher M Behrendt CA Cavus E Multi-organ assessment in mainly non-hospitalized individuals after SARS-CoV-2 infection: the Hamburg city health study COVID programme. *Eur Heart J.* (2022) 43:1124–37. 10.1093/eurheartj/ehab914 34999762 PMC8755397

[25] Abe N Tomita K Teshima M Kuwabara M Sugawa S Hinata N Distribution of cardiac troponin I in the Japanese general population and factors influencing its concentrations. *J Clin Lab Anal.* (2018) 32:e22294. 10.1002/jcla.22294 28763113 PMC5888119

[26] Shi S Qin M Shen B Cai Y Liu T Yang F Association of cardiac injury with mortality in hospitalized patients with COVID-19 in Wuhan, China. *JAMA Cardiol.* (2020) 5:802–10. 32211816 10.1001/jamacardio.2020.0950 PMC7097841

[27] Zhang L Wei X Wang H Jiang R Tan Z Ouyang J Cardiac involvement in patients recovering from delta variant of COVID-19: a prospective multi-parametric MRI study. *ESC Heart Fail.* (2022) 9:2576–84. 10.1002/ehf2.13971 35560820 PMC9288765

[28] Lala A Johnson KW Januzzi JL Russak AJ Paranjpe I Richter F Prevalence and impact of myocardial injury in patients hospitalized With COVID-19 infection. *J Am Coll Cardiol.* (2020) 76:533–46. 10.1101/2020.04.20.20072702 32517963 PMC7279721

[29] Korte W Buljan M Rösslein M Wick P Golubov V Jentsch J SARS-CoV-2 IgG and IgA antibody response is gender dependent; and IgG antibodies rapidly decline early on. *J Infect.* (2021) 82:e11–4. 10.1016/j.jinf.2020.08.032 32853597 PMC7445467

[30] Mahajan S Kunal S Shah B Garg S Palleda GM Bansal A Left ventricular global longitudinal strain in COVID-19 recovered patients. *Echocardiography.* (2021) 38:1722–30. 34555203 10.1111/echo.15199 PMC8653213

[31] Hayama H Ide S Moroi M Kitami Y Bekki N Kubota S Elevated high-sensitivity troponin is associated with subclinical cardiac dysfunction in patients recovered from coronavirus disease 2019. *Glob Health Med.* (2021) 3:95–101. 10.35772/ghm.2021.01025 33937572 PMC8071685

[32] Çakmak Karaaslan Ö Özilhan MO Maden O Tüfekçioǧlu O. Prevalence of cardiac involvement in home-based recovered coronavirus disease 2019 (COVID-19) patients: a retrospective observational study. *Ir J Med Sci.* (2021) 191:2057–62. 10.1007/s11845-021-02824-8 34714493 PMC8554733

[33] Kochi AN Tagliari AP Forleo GB Fassini GM Tondo C. Cardiac and arrhythmic complications in patients with COVID-19. *J Cardiovasc Electrophysiol.* (2020) 31:1003–8. 10.1111/jce.14479 32270559 PMC7262150

[34] Bürgi JJ Rösslein M Hornung H Jentsch J Boller VL Dollenmaier G Divergent humoral responses in mild to moderate SARS-CoV-2 infection over time – indication of persistence of the virus? *J Infect.* (2022) 84:418–67. 10.1016/j.jinf.2021.11.001 34748825 PMC8570801

[35] Hammarsten O Ljungqvist P Redfors B Wernbom M Widing H Lindahl B The ratio of cardiac troponin T to troponin I may indicate non-necrotic troponin release among COVID-19 patients. *Clin Chim Acta.* (2022) 527:33–7. 10.1016/j.cca.2021.12.030 34998858 PMC8744390

[36] Daniel C Sydney S Sabrina R Alison G Joon-Yong C Manmeet S SARS-CoV-2 infection and persistence throughout the human body and brain. *Nat Portf.* (2022). 10.21203/rs.3.rs-1139035/v1

[37] Ghugre NR Orbach A Biswas L Connelly KA Chan A Strauss BH Suspected subclinical myocarditis detected by cardiac magnetic resonance imaging late post COVID-19 recovery. *J Cardiol Cases.* (2021) 24:203–5. 10.1016/j.jccase.2021.04.014 34007346 PMC8120449

